# ANALYSIS OF SHOULDER ROTATOR TRAINING BY ARM WRESTLERS

**DOI:** 10.1590/1413-785220263401e296294

**Published:** 2026-02-13

**Authors:** Luiz Leopoldo Silva Gonzaga, Mauro Alexandre Pascoa, Paulo Roberto Moreira, André Luís Lugnani de Andrade, Maurício Etchebehere

**Affiliations:** 1 Universidade de Campinas (UNICAMP), Faculdade de Ciencias Medicas, Departmento de Ortopedia, Reumatologia e Traumatologia, Campinas, Sao Paulo, SP, Brazil.; 2 World Armwrestling Federation (WAF), Health and Performance Scientific Committee, Sofia, Bulgaria.; 3 Universidade de Campinas (UNICAMP), Faculdade de Ciencias Medicas, Laboratorio de Crescimento e Desenvolvimento, Centro de Investigacao em Pediatria, Sao Paulo, SP, Brazil.; 4 Faculdade Anhanguera Educacional, Fisioterapeuta, Campinas, Sao Paulo, SP, Brazil.

**Keywords:** Physical Functional Performance, Muscle Strength Dynamometer, Shoulder, Resistance Training, Sports Medicine, Desempenho Físico Funcional, Dinamômetro de Força Muscular, Ombro, Treinamento Resistido, Medicina Esportiva

## Abstract

**Introduction:**

Armwrestling is officially recognized in 160 countries affiliated with the World Armwrestling Federation. The movements involve the upper limbs, with emphasis on shoulder rotators, elbow flexors and extensors, and wrist.

**Objective:**

To evaluate the balance of agonist/antagonist forces in shoulder rotation and to verify differences in relation to the normative value of 64% for strength and 66% for power in the ratio between internal and external rotators.

**Methods:**

Seventeen international-level athletes were selected. A Biodex isokinetic dynamometer was used to evaluate the balance in peak torque of agonists and antagonists in shoulder rotation with strength and power tests.

**Results:**

Significant differences were found in the agonist/antagonist ratio in the power tests, with an average of 57% (P-value = 0.001) versus 66% of the normative value, and in the strength test, 58% (P-value = 0.064) versus 64% of the normative value.

**Discussion:**

The muscular strength and power of the subjects were analyzed in relation to normative parameters for internal and external shoulder rotation. The results were significant in relation to the normative values, indicating muscular imbalance in this group of athletes.

**Conclusion:**

The results indicate that the training performed by armwrestlers promotes a greater gain in strength of the internal rotators of the shoulder than their antagonists. ***Level of Evidence I; Diagnostic criteria previously tested on consecutive patients (with a universally applied "gold standard").***

## RESUMO

**Introdução:**

A Luta-de-Braço é reconhecida em 160 países filiados à Federação Mundial. Os movimentos envolvem membros superiores, com destaque para rotadores de ombro, flexores e extensores de cotovelo e punho.

**Objetivo:**

Avaliar o equilíbrio das forças agonistas/antagonistas na rotação do ombro e verificar diferenças em relação ao valor normativo de 64% para força e 66% para potência na razão entre rotadores internos e externos.

**Métodos:**

Foram selecionados 17 atletas de nível internacional. Utilizou-se um dinamômetro isocinético Biodex para avaliar o equilíbrio no pico de torque dos agonistas e antagonistas na rotação de ombro com testes de força e potência.

**Resultados:**

Foram encontradas diferenças significativas na razão agonista/antagonista nos testes de potência com média 57% (valor-P= 0.001) contra 66% do valor normativo e no de força 58% (valor-P= 0.064) contra 64% do valor normativo.

**Discussão:**

A força e potência muscular dos avaliados foram analisados em relação a parâmetros normativos para rotação interna e externa de ombro. Os resultados se revelaram significativos em relação aos valores normativos, indicando desequilíbrio muscular neste grupo de atleta.

**Conclusão:**

Os resultados indicam que o treinamento realizado por praticantes de luta-de-braço promove um ganho de força dos rotadores internos de ombro maior que seus antagonistas. ***Nível de Evidência I; Teste de critérios diagnósticos desenvolvidos anteriormente em pacientes consecutivos (com padrão de referência "ouro" aplicado).***

## INTRODUCTION

Arm-fighting is a force-fighting sport, a form of fighting between two opponents where there is no draw. According to historians of the World Arms Federation, papyrus was found in Egypt, more than 2,000 years before Christ, showing activities similar to arms struggle. Other works demonstrate the practice of this sport by the Vikings in Scandinavia and various artistic reports in other parts of the world. Currently it is a popular sport, but few people know it is officially recognized by the International Olympic Council and that it has a world federation (*World Armwrestling Federation*) with 160 affiliated countries. In Brazil, wrestling began to be practiced as a sport in 1949 at an event promoted by the school Ateneu Paulista in Campinas. In the 1960s there were tournaments promoted by the Paulistani newspaper Gazeta Esportiva where the campers Hugues Jorge, Nivaldo Félix, Washington Lázaro, the twins Celso and Célio Capelli among others also from Campinas were promoted.^1^ At the 2022 World Cup held in Antalya, Turkey, about 1500 athletes from 51 countries participated.^2^

In this sport are required mainly, much of the musculature of the chest and upper limbs especially the shoulder rotating groups, flexors and extenders of the elbow and wrist. The most experienced athletes outperform their opponents because they can maintain a greater amount of muscles contracted simultaneously, this process is called intermuscular coordination. The goal is to unite all the forces in a single vector. Among these movements is the internal shoulder rotation that is carried out by the muscles: chest,^3^ deltoid (front portion), large dorsal, larger round and subescapular; The antagonist movement, the external rotation of shoulder, is carried out by the muscles: intra-spinal, minor round and deltoid (back portion).^4^

The main wrestling techniques called "cross" and "up" consist of stabilizing the inner rotation of the shoulder while the athlete performs the elbow flexion beyond all strength in the wrist, which is required to master the "top" of the opponent's lever, at the same time a lateral bend of the trunk is performed to "open the set" and lower the opponent's hand to win the fight.^5^

The aim of this study is to evaluate the proportion of the agonist (internal rotation) and antagonist (external rotation) forces in the shoulder rotation movement of wrestling practitioners.

## MATERIALS AND METHODS

A cross-sectional study was conducted to test the dominant limbs of athletes with an isokinetic dynamometer ^5^ (Biodex System 4 Pro from Biodex Medical System, Inc. 20 Ramsey Road Shirley, New York) in a concentric/concentric assessment of the balance of shoulder rotation agonists/antagonists.

All have completed the Terms of Free and Informed Consent – TCLE. The study was approved by the Ethics and Research Committee Number of the CAAE: 76328317.9.0000.5404

### Inclusion Criteria

- Male adults, over 18 years of age and at least 5 years of wrestling practice;
- International competitive level, only Brazilian champions and vice champions.

### Exclusion criteria

Present functional score DASH^6^ greater than 39, where less than 20 is excellent, 20 to 39 is good, 40 to 60 regular and above 60 bad (functional disability), that is, were excluded those who had regular and bad score, being accepted only those rated as excellent and good.

Show pain or discomfort before or during tests.

### Isocinetic Assessments

Athletes were allowed to perform body warming according to each individual's habit. We provided varied and elastic weights, such as those used by athletes in the heating of training and championships, before being positioned on the equipment.

As a protocol, the evaluation on the isocinetic dynamometer promotes familiarity with the same speeds that will be tested, but with fewer repetitions, and is initiated by the force test with movements performed at 60o/s and for the shoulder power test at 180o/s.^7,8^ Between familiarity with the equipment and the main test as well as between the variations in the force to power test, a 90-second interval was observed for each series of movements. The tests were performed sitting to stabilize the athlete's body and isolate the shoulder movement without influence from other muscle groups that were not being tested.

The dominant arm was positioned with the shoulder abducted 45 degrees, the elbow and forearm supported on support fixed on the lever and hand holding manople that kept the handle in neutral position without flexo-extension or prono-supination. The machine axis was positioned 20 degrees of vertical rotation and 50 degrees of horizontal rotation and the height of the chair was adjusted with the elbow supported without lifting or depressing the scapula.^9^

The evaluations were performed with concentric strength tests, which is the strength that shorten the muscle generating movement, in all variations to extract the difference between the agonist (internal rotators) and antagonist (external rotators) muscles involved in the joint movement tested.

### Statistical Methodology

To describe the profile of the sample for the variables in study were made frequency tables of the categorial variables (age, height, weight, BMI, forearm length and practice time), with absolute frequency values (n) and percentage (%), and descriptive statistics of the numerical variables of the peak torque, found in the evaluation of external/internal shoulder rotation movements with strength and potency test, mean values, standard deviation and confidence interval.^10^

The data that best demonstrate the differences between the agonist and the antagonist and their variations in the joint movements involved were selected to evaluate results: the peak torque that has its result expressed in Nm (newton-meter) and the agonist/antagonist ratio of the forces in these movements found by dividing the peak torque of the agonist by the peak torque antagonist and multiplied by 100 expressed in % (percentage).^5^

To compare the results of the athletes with the normative values stipulated by the equipment, the hypothesis test for average was used and the confidence level of the sample (n=17) was validated by the confidence interval of 95%.^11^ The level of significance adopted for the statistical tests was 5%.^8^

## RESULTS

17 athletes were selected for the tests. Table 1 shows the characteristics of the total sample (n=17). The average age of the participants was 36 years. Table 2 presents the performance of athletes in terms of peak strength in external and internal rotations with values expressed in Newton per meter (Nm), with these values being calculated the ratio between agonist and antagonist, which present significant values or not.

**Table 1:** Descriptive analysis of the evaluated group.

Variable	N	Media	SD	IC 95%
Age (years)	17	36	8.42	(31.6; 40.3)
Height (cm)	17	180	0.08	(176; 184)
Weight (kg)	17	95.7	19.6	(85.7; 105.8)
BMI	17	29.5	4.89	(26.9; 32.0)
Frontarm (cm)	17	50.2	2.15	(49.1; 51.3)
Practice Time (years)	17	19.3	8.28	(15.1; 23.6)

95% CI: 95% confidence interval of the average.

**Table 2:** Athletes’ performance on the peak torque of the external and internal shoulder rotations.

Variable	N	Media	SD	IC 95%
External Rotation Force	17	58.9Nm	13.6	(52; 66)
Internal Rotation Force	17	102.7Nm	22.8	(91; 114)
External Rotation Power	17	55.2Nm	12.2	(49; 61)
Internal Rotation Power	17	96.8Nm	18.6	(87; 106)

95% CI: 95% confidence interval of the average.

Table 3 shows separately the comparisons of the values found in the evaluation of the athletes with the normative parameters in the difference in peak torque, the agonist/antagonist ratio both in the strength test, which was not significant with value-P = 0.064, and the power, which was significant with value-P = 0.001, with their differences expressed in percentage (%).

**Table 3:** Comparison of athlete parameters with the normative values of the agonist/antagonist ratio.

Variable	N	Media	SD	Normative value	IC95%	P-value
test strength	17	58.6%	11.2	64 %	(53; 64)	P=0.064
Power test	17	57.4%	8.8	66 %	(53; 62)	P=0.001

* P-value referring to the hypothesis test for average for comparison with the normative values. 95% CI: 95% confidence interval of the average.^10–12^

## DISCUSSION

This study analyzed the strength and muscle potency of athletes in relation to normative parameters for internal and external shoulder joint rotation of international-class wrestling practitioners. The internal and external rotations were evaluated as fundamental for the execution of a good technique in this sport.

The muscles are contracted in different functions, here they have been analyzed as agonists and antagonists. The agonists are the muscles responsible for the desired action while the antagonists simultaneously contract in opposition and relax to allow a smooth movement.^13^

In high-performance athletes and especially in this sport of strength and power, the peak torque is crucial in the result of the fight. For the parameters (spike torque and agonist/antagonist torque ratio) the evaluation on the isokinetic dynamometer is very accurate and these were the data analyzed in this study, also considering that this force difference is used to demonstrate the balance of the muscles that act in the joints.^5^

"*Isocinetic dynamometers are measuring instruments that provide clinicians with information about the dynamics, i.e., movement, mechanical performance of muscle groups*" Dvir.^14^

The shoulder rotators were tested with the sole aim of observing a possible change in the agonist/antagonist ratio because in this sport the internal rotation is much more demanding.^14^

According to the results, the difference between the sample parameters and the normative values for agonist/antagonist ratio in the assessment of shoulder rotation was significant in the power test at 180 degrees per second, according to the data P=0.001 and no significant difference with P=0.064 in the strength test at 60 degrees per second as demonstrated in Table 3.

The normative values used in this work, from 64% to 66% agonist/antagonist ratio in strength and potency tests respectively, are the reference values of the Biodex System isokinetic dynamometer that coincide with an approximate average of the values found by Brown et al. (1988) 61% a 72% in male athletes, Connelly et al. (1989) 62% in the test with male average audience, Ellenbecker (1988) 65% to 72% for male athletes, McMaster et al. (1991) 55% to 78% for the general public and male athletes and Reid et al. (1989) 53% to 66% for the general public and male athletes respectively.^15^

The ratio between the internal and external rotor forces found in wrestling athletes is different from normative values, but even greater differences were found in judo player as demonstrated by Marcondes et al. (2019) where the percentage difference in the shoulder rotators tests of the dominant arms was 49.4% for strength and 42.9% for power, according to the isokinetic assessment for the shoulder rotators.^16^ Re-evaluating training is fundamental in the pursuit of a better balance of forces and with that, a possible improvement in performance in this sport.

## CONCLUSION

The results of the evaluations of shoulder rotators in wrestling practitioners show, by analysis of performance at peak torque of the agonists and antagonists of this movement, a lower power of the external rotators relative to the internal ones.

## Data Availability

The data set of this article is available in Mauro CIPED - It is necessary to publish in REDU UNICAMP, via the login of the guide: https://www.sbu.unicamp.br/sbu/repositorio-de-dados-de-requisa-da-unicamp/. Source: NORMATIVA_PRPG_CCPG_1_2024.
